# Soil-Transmitted Helminth infections reduction in Bhutan: A report of 29 years of deworming

**DOI:** 10.1371/journal.pone.0227273

**Published:** 2020-01-03

**Authors:** Tshering Dukpa, Nidup Dorji, Sangay Thinley, Wangchuk, Karma Tshering, Kinley Gyem, Diki Wangmo, Passang Lhamo Sherpa, Tshering Dorji, Antonio Montresor

**Affiliations:** 1 Faculty of Nursing and Public Health, Khesar Gyalpo University of Medical Sciences of Bhutan, Thimphu, Bhutan; 2 Comprehensive School Health Programme, Department of Public Health, Ministry of Health, Thimphu, Bhutan; 3 Department of Microbioloy, Jigme Dorji Wangchuk National Referral Hospital, Thimphu, Bhutan; 4 Royal Center for Disease Control, Thimphu Bhutan; 5 Laboratory Unit, Trashigang District Hospital, Trashigang, Bhutan; 6 Department of Control of Neglected Tropical Diseases, World Health Organization, Geneva, Switzerland; UMASS Medical School, UNITED STATES

## Abstract

Soil Transmitted Helminth (STH) infections affect over 1.5 billion people worldwide. Although prevalent in all age groups, school aged children are a high-risk groups for STH infections. In Bhutan, epidemiological data on STH were collected from western Bhutan in 2003, which found a prevalence of 16.5%. However, little evidence is available on the prevalence of infection at national level. Therefore, this study was conducted with the aim to assess the prevalence and intensity of STH infections, and identify significant correlates of STH among students. A school-based survey was conducted in three regions of Bhutan. Two-stage cluster sampling was adopted to select a sample of 1500 students from 24 schools, in equal proportion from three regions of the country. A total of 1456 (97%) students were interviewed and their stool sample examined for the presence of parasites. Mini-FLOTAC technique was used to detect the parasite eggs/ova. The prevalence of any STH infection was 1.4%, with 0.8% *Ascaris lumbricoides*, 0.5% *Trichuris trichiura* and 0.2% hookworms. The eastern region had the highest prevalence at 2.3%. Except for one student who had moderate intensity of *A*. *lumbricoides*, the rest had light infection. Any STH presence was significantly associated with father’s occupation, father’s education level, type of house and the flooring of the house in which students reported to live. No significant associations were observed between water, sanitation and hygiene (WASH) variables measured and presence of any STH infection. The prevalence of STH was found to be very low with primarily light intensity in this study. Nonetheless, it was also found that the sanitation situation is not ideal in the country, with several students reporting constant or partial open defecation leading to environmental contamination. Based on this prevalence and in line with the WHO guideline, it is recommended that deworming be reduced to once a year in combination with concerted health education on proper hygiene and sanitation practice.

## Introduction

Soil Transmitted Helminth infections (STH) are among the most common infections in low and middle-income countries [1, 2]. There are four important species of STHs that infect humans: *Ascaris lumbricoides* (roundworm), *Trichuris trichiura* (whip-worm) and *Ancyclostoma duodenale and Necator americanus* (hookworms)[3, 4]. Global estimates report 804 million people infected with roundworm, 477 million with whipworm and 472 million with hookworms [5]. These parasites are transmitted through contamination of soil by human feces containing eggs, and subsequently acquired by accidental ingestion or through skin penetration as in the case of hookworm larvae [3, 6]. Thus, they mostly affect children living in underprivileged communities with poor sanitation and hygiene or inadequate access to safe and clean water [1–3, 7, 8].

STH infections rarely result in death, but increasing evidence suggests that STH infection in children are associated with impairment of physical growth and mental development and micronutrient deficiencies including iron deficiency anemia, leading to poor learning ability and school absenteeism [3, 6, 9, 10]

Currently, the control method for STH recommended by the World Health Organization (WHO) is preventive chemotherapy [6]. The current strategy involves treating school-aged children (5-14years old) and pre-school children (2–4 years old) regularly with albendazole or mebendazole irrespective of their infection status.

WHO had set a target to eliminate morbidity related STH infection by 2020. This can be achieved by regularly treating 75% of the children in endemic areas [9]. In addition, the combined effect of PC, provision of clean water, and improved sanitation coupled with behavioral changes are found to be effective in preventing re-infection [6, 9–12].

**Fig 1:**
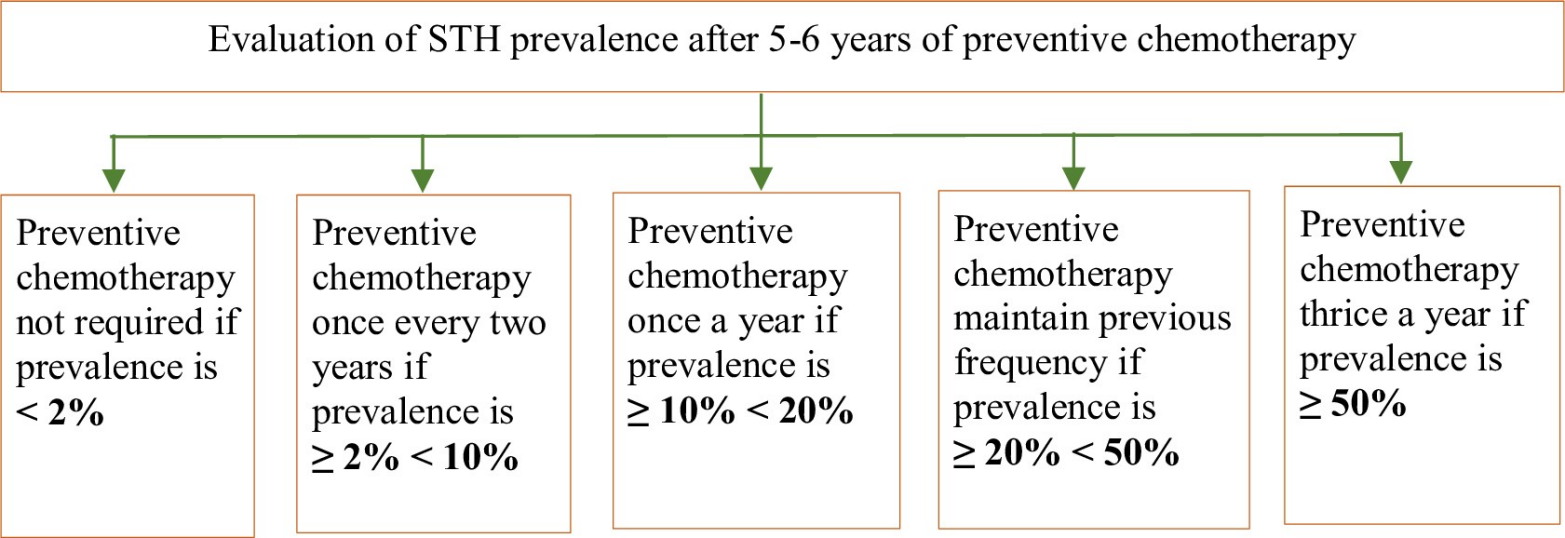
Decision tree for administration of preventive chemotherapy in treated population. Adapted from Helminth control in school-age children: a guide for managers of control programmes, World Health Organization, 2011.

After several years on preventive chemotherapy, the prevalence can be significantly reduced, but in absence of appropriate sanitation infrastructure, if the intervention is interrupted, there is a rapid rebound of STH prevalence to the initial levels [6]. To avoid this occurrence, WHO established a second set of threshold to be applied to epidemiological data collected after 5–6 years of preventive chemotherapy as presented in Fig 1.

Bhutan introduced a school deworming program in 1988 [13]. Currently, a single dose of albendazole is administered every six months to students through the school-based program. Pre-school children are given deworming medicine every six months through the health care system, and any individual suspected of having helminth infection are treated free of charge. Records with the Ministry of Health, Bhutan indicated that deworming coverage for school children ranged from 80 to 98 percent since 2003.

In addition to deworming, the country has progressed in terms of socio-economic development, enhancing the living conditions of the people. It is thus expected that the STH infections would have decreased over the years. A few studies on STH conducted in 1985, 1986 and 1989 found STH prevalence between 20% to 70% [13]. As presented in Table 1, the study in 2003 among schools in western Bhutan found a prevalence of 16.5% [13]. However, this information was not generalizable to effect policy change related to STH control for the country. Therefore, the aim of this study was to assess the nationwide prevalence of STH infection among students, and examine the correlates of STH infection to inform in reviewing policy related to STH control for the country.

**Table 1:** Summary findings from 2003 STH study among students in western Bhutan.

	Total sample(n = 266)		Schools treated in the last three months (n = 104)		Schools not treated in the last three months (n = 162)	
	Prevalence	Moderate heavy intensity	Prevalence	Moderate heavy intensity	Prevalence	Moderate heavy intensity
*Ascaris lumbricoides*	12.8%	3.0%	1.9%	1.3%	19.8%	4.0%
*Trichuris trichiura*	5.6%	-	2.9%	--	7.4%	--
Hookworm	1.1%	-	-	--	1.9%	--
Prevalence of any STH infection	16.5%	3.0%	4.8%	1.3%	24%	4.0%
*Taenia solium*	6.7%	NA	0	NA	11.0%	NA

Credit: Allen H, Sithey G, Padmasiri EA, and Montresor A. Epidemiology of soil-transmitted helminths in the western region of Bhutan. The Southeast Asian journal of tropical medicine and public health. 2004;35(4):777–9

## Materials and method

### Study setting and population

Bhutan is a small landlocked country, covering an area of 38,394 Km^2^ in the eastern Himalayas with a population of 727,145. It shares its border with Tibet part of China in the north and India in the east, west and south. The country is mainly mountainous with elevation ranging from as low as 160 meters above sea level in the hot and humid southern foothills, to 7,314 meters snowcapped alpine Himalayas in the north. It is administratively divided into three regions as Eastern, Central and Western Bhutan.

**Fig 2:**
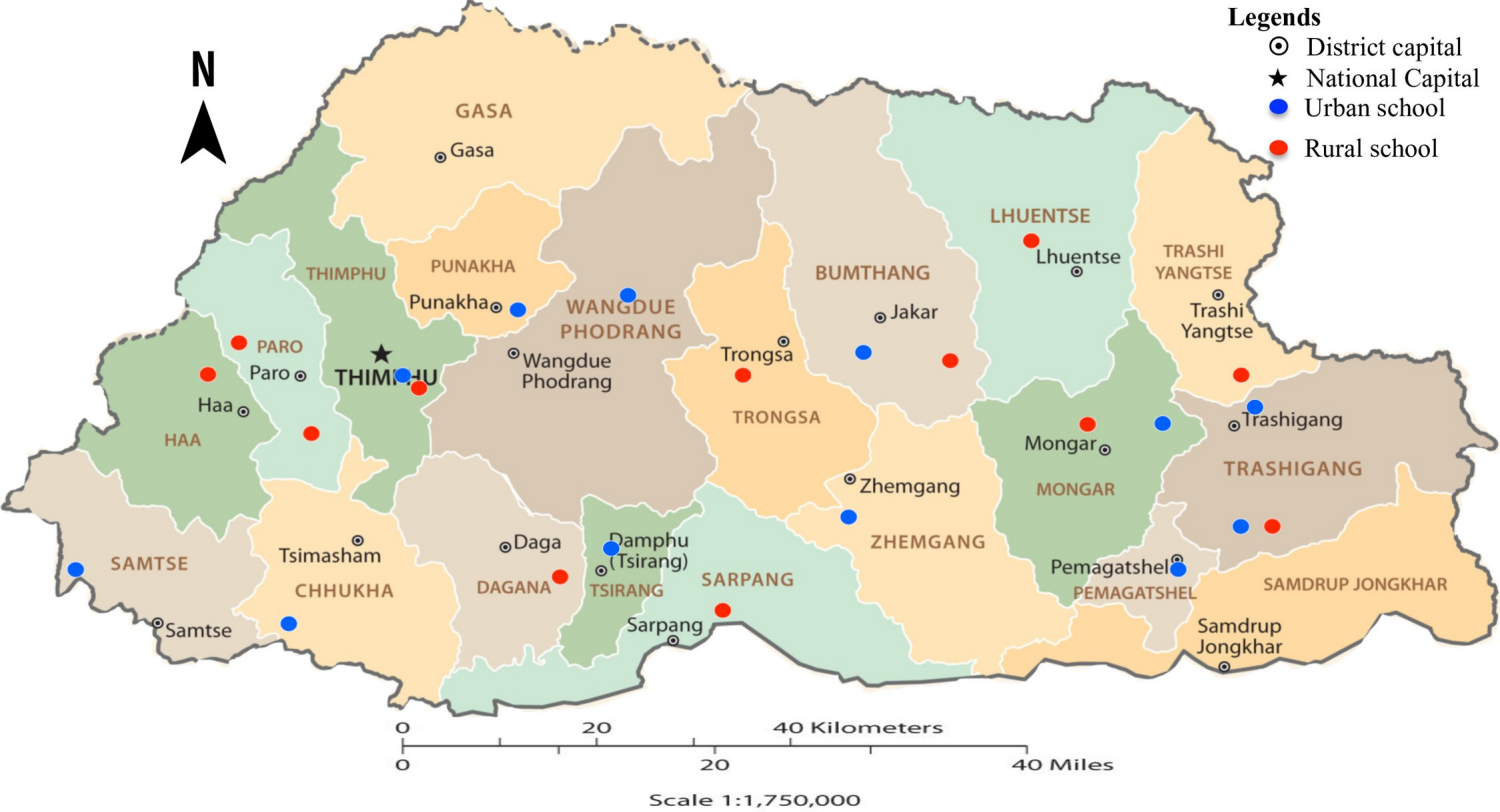
Location of schools sampled from different regions of Bhutan, 2017. Adapted from The World Factbook–Bhutan, Central Intelligence Agency, 2019.

This study was conducted among students from both urban and rural schools in three regions (east, west and central) of Bhutan as shown in Fig 2, between June and September 2017. In these regions, school students from grade 3 to 8 were included in the study. This grade range was chosen as students in this grade fall in the age group at high risk for STH infection [6, 9] and also because they were old enough to respond to the questionnaire administered to understand the correlates of STH infection.

### Study design and sample estimation

This was a school based cross-sectional study. The sample size for this study was calculated using single proportion formula with an estimated prevalence of 16.5% based on a previous study, 95% confidence interval (CI) level (Z (1-ά/2) = 1.96), 3.2% margin of error, the minimum required sample size was 517. To minimize errors related to cluster sampling, a design effect of 2.6 was considered based on similar studies in the region [14, 15]. Further, an attrition rate of 10% was added to the sample. Thus, the final sample size obtained was 1500.

The estimated sample was divided equally among the three regions. A two-stage cluster sampling technique was adopted to select schools and students in each region. In the first stage, 8 schools (four each from urban and rural settings) were randomly selected from a list of schools maintained by the Ministry of Education (MoE) for the year 2017. The selection of 8 schools was based on the inclusion of 10 students each from grade 3 to 8 leading to recruitment of 60 students in total to represent each selected school. The second stage involved the selection of students from each school.

A random number table in Excel was used in selecting both the schools and the students within each grade. In the case where a selected student was unable to participate, the student in the next random number list was recruited. Further, consideration to include 8 schools from each region was also based on the logistic feasibility and the budget availability for the study. Thus, 24 schools were included from three regions for the study.

#### Inclusion criteria and exclusion criteria

Students from grades 3–8 whose parents/guardians/teachers signed a written consent and those who gave verbal assent were included in the study. Students in grades 9 and 10 were not included, despite their similarities with those in grade 8 in terms of infection risk, because of the school structure in Bhutan. The common division of grades in Bhutanese schools are primary (preparatory to grade 6), middle secondary (preparatory to grade 8), higher secondary (grade 9 to 12) and a few central schools (preparatory to grade 12). Since the study design required 60 students from each school, including students from higher secondary schools would have limited the inclusion of students at higher risk in grades 3 to 5.

Similarly, students who were cognitively impaired, had an anti-helminthic drug within the past six months, did not provide stool samples, and those in private schools were also excluded. Students in private schools were excluded because there were only few private schools with enrollment from grade 3–8 and most of these were concentrated in one region and in urban settings. It was also felt that inclusion of private schools might compromise smooth conduct of the study within the available time and financial resources, since the Ministry of Education doesn’t have administrative and management oversight of these schools.

### Data collection

Six teams each consisting of two health assistants, one laboratory technician, and a supervisor, who was a member of research team, conducted the data collection. All members received three days of training to ensure consistency among the teams.

A pre-tested structured questionnaire was used to collect socio-demographic information and factors associated with STH infection based on water, sanitation, and hygiene (WASH) behavior of the students. Prior to data collection, an information letter was sent to the sampled schools inviting their participation. Two days were spent in collecting data from each school. On day one, the team met with the principal, parent/guardian/teacher and obtained informed consent. Then the students were interviewed after informing them about the study and obtaining verbal assent for participation.

#### Sample collection and analyses

Following the interview, the laboratory technician in the team explained and demonstrated on how to collect stool sample. Each student was provided with a clean screw capped plastic container with attached scoop that was labeled with the student’s name, identification number, date, and time of collection of the specimen. Students were asked to spread a clean sheet of plastic wrap/paper on the toilet floor, defecate on it to avoid contamination with urine, water or soil; collect 2–3 scoops of specimen in the container, re-cape it tightly to prevent leakage and place it inside a zip-locked plastic bag before returning it to the research team.

The stool samples were collected on the same day or on the following day. At the collection site, immediately after receiving the sample, 2 grams from each sample was transferred to the respective pre-labeled fill-FLOTAC, where it was fixed and homogenized in 2 ml of 5% formalin (dilution 1:1) before transporting it to the respective testing centers for examination.

There were two testing centers set up in each region manned with two-trained laboratory technicians and a laboratory supervisor from the study team. The samples were analyzed within one week from the date of collection.

At the testing center, the helminth ova were detected using mini-FLOTAC, a microscopic diagnostic technique, which is sensitive and appropriate for preserved stool [16, 17]. The fill-FLOTAC containing fixed fecal sample was homogenized further with addition of 38 ml of flotation solution (FS2) (dilution 1:20). Then, each mini-FLOTAC chamber was filled with 1 ml fecal suspension from the fill-FLOTAC. The loaded mini-FLOTAC was allowed to stand for 10 minutes for the eggs and cysts to float. After 10 minutes, it was examined for helminth eggs/ova under a microscope by trained laboratory technicians.

Mini-FLOTAC is one of the diagnostic methods suggested by WHO [18]. The method has sensitivity similar to the Kato-Katz [19] and has the advantage that the laboratory examination can be conducted within 2 weeks from the collection of the specimen(differently from the Kato-Katz that must be conducted within few hours), which simplifies the logistic.

The laboratory supervisor confirmed the egg detection on each slide under a microscope and checked every negative slide. Further, the laboratory supervisors from different testing centers crosschecked random slides to ensure quality control. In addition to STH parasites, other parasites were also detected and recorded.

### Data management and analysis

Data were checked for completeness on hard copies by the investigators before entry into EpiData version 3.1. The data were double entered and compared with the original keyed-in data to detect and correct data entry errors. It was then transferred into Stata^®^ version 12.1 (2008–2011) StataCorp LP, College Station, TX,USA), where it was cleaned and analyzed.

Descriptive statistical methods were used to summarize the data. The prevalence of any STH infection was calculated as the ratio of number of students found positive for any STH species to the total number of students who provided complete data. Similarly, prevalence was also reported separately by STH species. Intensity of infection, based on parasite-specific egg counts was determined by multiplying the egg count by 10 to adjust the eggs per gram (epg) of feces. The intensity was categorized as light, moderate or heavy intensity following WHO recommended thresholds [6] as shown in Table 2.

**Table 2:** Classification of intensity of soil transmitted helminths infection ^a^.

Parasite	Light-intensity Infection ^b^	Moderate-intensity infection ^b^	Heavy-intensity infection ^b^
*A*. *lumbricoides*	1–4 999 epg	5000–49 999 epg	≥ 50000 epg
*T*. *trichiura*	1–999 epg	1000–9 999 epg	≥ 10000 epg
Hookworms	1–1 999 egp	2000–3 999 epg	≥ 4000 epg

a Adapted from: World Health Organization. Helminth control in school-age children: a guide for managers of control programmes, World Health Organization; 2011.

b epg = eggs per gram of feces

Pearson’s Chi-square test and Fischer exact test as appropriate were performed to examine any association between STH infection and independent factors. The education level of father and mother was recoded into five levels (illiterate, primary, high school, college/university and others). Likewise, the occupation of father and mother was recoded into three levels (farmer, salaried and others). The flooring type was also recoded into three levels (mud, concrete/tile and others) since concrete and tile flooring are of similar nature. A *p*-value of <0.05 was considered to look for any significant associations.

### Ethical considerations

The study protocol was approved by the Research Ethics Board of Health (REBH) vide approval letter no REBH/Approval/2017/033 and permissions were obtained from the study sites prior to data collection. The participation of student was voluntary. Written informed consent was obtained from the parent/guardian/teacher. Assent was also taken from the student. The data were kept confidential and securely stored with access available only to the members of the research team. Students with positive results were informed and referred to the health facility for appropriate treatment as per the treatment guidelines of the Ministry of Health, Bhutan.

## Results

### Socio-demographic characteristics of the sample

A total of 1456 students (97%), comprising 484 from East, 474 from West and 498 from Central region participated in the present study. As reflected in Table 3, males comprised 52.4% and females 47.2% of the total participants. Age of the students ranged from 7 to 20 years with the mean age of 11.9 (SD = 2.2) years. The majority (78.8%) of the students were day-scholars. Forty seven percent of the students were of Sharchop ethnicity. About half (48.2%) of students’ mothers and 41.6% of fathers were farmers. Less than half (40.2%) of the mothers were illiterate and 34.2% of the mothers had primary education. Over one fourth (26.9%) of students’ fathers were illiterate and 49.6% of the fathers had primary education. Residing in a stone house was reported by 39.6% of the students and 12.4% mentioned living in a mud house. More than half (55.1%) reported ‘others’ category as the floor type in their house, which included 99.6% wooden plank and rest bamboo plank flooring. Very few (5.1%) students reported houses with mud flooring.

**Table 3:** Socio-demographic characteristics of students in the sample investigated for STH in Bhutan, 2017 (n = 1456).

Socio-demographic characteristics	Measurement	n	(%)
Age	<12 years	669	(45.9)
	≥12 years	787	((54.1)
Mean = 11.9	SD = 2.2	Median = 12.0	Min-Max = 7–20
Sex	Male	693	(47.6)
	Female	763	(52.4)
Grade	grade 3	237	(16.3)
	grade 4	240	(16.5)
	grade 5	246	(16.9)
	grade 6	246	(16.9)
	grade 7	242	(16.6)
	grade 8	245	(16.8)
Ethnicity	Ngalop	293	(20.1)
	Sharchop	689	(47.2)
	Lhotsam	252	(17.2)
	Kheng-Bumthap	222	(15.3)
Father’s occupation	Farmer	605	(41.6)
	Salaried	704	(50.8)
	Don’t know	111	(7.6)
Mother’s occupation	Farmer	702	(48.2)
	Salaried	734	(50.4)
	Don’t know	20	(1.4)
Father’s education	Illiterate	392	(26.9)
	Primary school	359	(24.7)
	High school	231	(15.9)
	College/ University	131	(9.0)
	Others ^a^	343	(23.6)
Mother’s education	Illiterate	585	(40.2)
	Primary school	280	(19.2)
	High school	183	(12.6)
	College/ University	35	(2.4)
	Others ^a^	373	(25.6)
School type	Lower Secondary School	787	(54.1)
	Middle Secondary School	179	(12.3)
	Higher Secondary School	59	(4.1)
	Central School	431	(29.6)
Type of student	Boarding student	309	(21.2)
	Day scholar	1147	(78.8)
House type	Hut	208	(14.3)
	Stone house	576	(39.6)
	Mud house	181	(12.4)
	Modern concrete	491	(33.7)
Flooring type	Mud flooring	74	(5.1)
	Concrete /tile flooring	580	(39.8)
	Others ^b^	802	(55.1)

^**a**^ Non-formal education, Monastic education, Don’t know

^**b**^ Bamboo, Planks

### Water, sanitation and hygiene (WASH)

The study also investigated the source of drinking water, use of toilets and personal hygiene related to hand washing. The responses were elicited using a questionnaire with a Likert scale: always (3), sometimes (2) and never (1). More than half (54.5%) of the students’ sourced water for drinking from the tap and 33.2% of the students reported drinking water sometimes from streams and rivers. Only 105 students (7.2%) sometimes used rainwater for drinking. In terms of drinking practices, 19.2% boiled and 17.5% always filtered water before drinking (Table 4).

**Table 4:** WASH behavioral characteristics of students in the sample investigated for STH in Bhutan, 2017 (n = 1456).

WASH items	Measurement	*n*	(%)
Drinking tap water	Never	18	(1.2)
	Sometimes	645	(44.4)
	Always	793	(54.5)
Drinking water from stream/ river	Never	966	(66.4)
	Sometimes	484	(33.2)
	Always	6	(0.4)
Drinking rain water	Never	1351	(92.8)
	Sometimes	105	(7.2)
	Always	0	(0.0)
Drinking water from pond	Never	1288	(88.5)
	Sometimes	168	(11.5)
	Always	0	(00.0)
Drinking boiled water	Never	83	(5.7)
	Sometimes	1093	(75.1)
	Always	280	(19.2)
Drinking filtered water	Never	320	(22.0)
	Sometimes	882	(60.6)
	Always	254	(17.5)
Cleanliness of toilet at school	Never	35	(2.4)
	Sometimes	1225	(84.1)
	Always	196	(13.5)
Using flush toilet	Never	20	(1.4)
	Sometimes	857	(58.9)
	Always	579	(39.8)
Using pit latrine	Never	803	(55.2)
	Sometimes	593	(40.7)
	Always	60	(4.1)
Practicing open defecation	Never	460	(31.6)
	Sometimes	984	(67.6)
	Always	12	(0.8)
Availability of water for hand washing at school	Never	4	(0.3)
	Sometimes	923	(63.4)
	Always	529	(36.3)
Availability of soap for hand washing at school	Never	153	(10.5)
	Sometimes	935	(64.2)
	Always	368	(25.3)
Washing hand before meal	Never	19	(1.3)
	Sometimes	822	(56.5)
	Always	615	(42.2)
Using soap while washing hand	Never	15	(1.0)
	Sometimes	1093	(75.1)
	Always	384	(23.9)
Washing hand after defecation	Never	32	(2.2)
	Sometimes	775	(53.2)
	Always	649	(44.6)
Engaging in agricultural work	Never	88	(6.0)
	Sometimes	1232	(84.6)
	Always	136	(9.3)
Wearing foot wear outside house	Never	62	(4.3)
	Sometimes	721	(49.5)
	Always	673	(46.2)
Washing fruits before consuming	Never	51	(3.5)
	Sometimes	906	(62.2)
	Always	499	(34.3)
Using spoon for eating food	Never	56	(3.9)
	Sometimes	972	(66.8)
	Always	428	(29.4)
Keeping nail short	Never	4	(0.3)
	Sometimes	633	(43.5)
	Always	819	(56.3)

Only 13.5% mentioned that toilets in school were always clean. Always using a flush toilet was reported by 39.8% of the students. More than half (67.6%) of students sometimes practiced open defecation. Less than half (33.3%) of the students reported always having water and 25.3% reported always having soap for hand washing at school. Less than half (42.2%) of the students always washed hands before meals and 44.6% did it after defecation. Indulging agriculture work sometimes was reported by 84.6% of the students. About half (46.2%) of the students mentioned always wearing footwear while outside house. More than half (56.3%) of the students always kept their nails short (Table 4).

### Prevalence and intensity of soil transmitted helminths

The overall prevalence of any STH infection was 1.4%.The prevalence was slightly higher in the eastern region (2.3%), followed by central region (1.4%) and lowest in western region (0.6%). The most detected STH was *A*. *lumbricoides* 11 (0.8%), followed by *T*. *trichiura* 8 (0.5%) and hookworms 3 (0.2%) (Table 5). Among the infected, only one student had double infections but no one had triple infections in this study. In addition, two students from western region and one student form central region were detected with tapeworm infection.

**Table 5:** Prevalence of soil-transmitted helminths infection among students in the sample investigated for STH in Bhutan, 2017 (n = 1456).

	Overall (n = 1456)		Eastern (n = 484)		Central (n = 498)**		Western (n = 474)	
	Number	Prevalence	Number	Prevalence	Number	Prevalence	Number	Prevalence
	Positive	(95% CI)	Positive	(95% CI)	Positive	(95% CI)	Number	(95% CI)
**Any STH**	21	1.4 (0.8–2.0)	11	2.3 (0.9–3.6)	7	1.4 (0.4–2.4)	3	0.6 (0.0–1.3)
***A*. *lumbricoides***	11	0.8 (0.3–1.2)	9	1.9 (0.7–3.1)	2	0.4 (0.0–0.9)	0	-
***T*. *trichiura***	8	0.5 (0.2–0.9)	2	0.4 (0.0–0.9)	3	0.6 (0.0–1.3)	3	0.6 (0.0–1.3)
**Hookworms**	3	0.2 (0.0–0.4)	0	-	3	0.6 (0.0–1.3)	0	-
Tapeworms*	3	0.2 (0.1–0.6)	0	-	1	0.2 (0.1–1.4)	2	0.4 (0.1–1.7)

*2 *Taenia solium* and 1 *Hymenolepis nana*

** one student had double infection (*A*. *lumbrioides & T trichiura*)

Except for one student, who had moderate infection of *A*. *lumbricoides* from Eastern region, all other students had light intensity infections. No heavy infections were detected among the students (Table 6).

**Table 6:** Intensity of soil-transmitted helminths infection among students in the sample investigated for STH in Bhutan, 2017 (n = 1456).

Types of helminthes	Overall (n = 1456)			Eastern (n = 484)			Western (n = 474)			Central (n = 498)		
	L n(%)	M n(%)	H n(%)	L n(%)	M n(%)	H n(%)	L n(%)	M n(%)	H n(%)	L n(%)	M n(%)	H n(%)
*A*. *lumbricoides* ^a^	10 (90.9)	1(9.1)	-	8 (88.9)	1 (11.1)	-	-	-	-	2 (100.0)	-	-
*T*. *trichiura* ^b^	8 (100.0)	-	-	2 (100.0)	-	-	3 (100.0)	-	-	3 (100.0)	-	-
Hookworms ^c^	3 (100.0)	-	-	-	-	-	-	-	-	3 (100.0)	-	-

^***a***^ Normal range: Light(L) = 1–4 999 epg; Moderate(M) = 5000–49 999epg; Heavy(H) = ≥50 000 epg

^***b***^ Normal range: Light(L) = 1–999 epg; Moderate(M) = 1000–9 999epg; Heavy(H) = ≥10 000 epg

^***c***^ Normal range: Light(L) = 1–1 999 epg; Moderate(M) = 2000–3 999epg; Heavy(H) = ≥4 000 epg

### Factors associated with soil transmitted helminths infection

Potential factors associated with STH infection were explored. The findings associated with socio-demographic characteristics are summarized in Table 7. No significant difference in the prevalence of any STH was observed between urban and rural settings. Prevalence of any STH was significantly associated with occupation (P = 0.022) and education level (P = 0.040) of students’ father, flooring type (P = 0.018) and type of house (P = 0.007) in which students reside. A higher number of infection were found in students whose fathers’ occupation was reported as farmers as compared to other occupation groups. The infection was also at higher rate among students who reported their fathers were illiterate than those in other groups. Similarly, infection was higher in those who reported the flooring type as ‘others,’ which mainly consists of wooden plank and bamboo flooring. Students who mentioned staying in stone houses had higher rate of infection compared to those who reported other types of houses. Similarly, STH infection was not significantly associated with WASH variables measured.

**Table 7:** Relationship between socio-demographic characteristics and presence of soil-transmitted helminthes among students in Bhutan, 2017(n = 1456).

Socio-demographic characteristics	Students Examined	Presence of any STH		
	n (%)	n (%)	χ^2^	p-value
Sex				
Female	763 (52.4)	11 (1.4)	0.00	0.998
Male	693 (47.6)	10 (1.4)		
Age				
<12 years	669 (45.9)	13 (1.9)	2.1845	0.139
≥ 12 years	787 (54.1)	8 (1.0)		
School setting				
Rural	731 (50.2)	13 (1.8)	1.1665	0.280
Urban	725 (49.8)	8 (1.1)		
Type of Student				
Boarding	309 (21.2)	5 (1.6)	0.0853	0.770
Day scholar	1147 (78.8)	16 (1.4)		
Father’s Occupation				
Farmer	605 (41.1)	15 (71.4)	-	0.022*
Salaried	740 (51.2)	6 (28.6)	-	
Don’t know	111 (7.7)	0 (0.0)	-	
Mother’s Occupation				
Farmer	702 (48.2)	15 (71.4)	-	0.107
Salaried	734 (50.4)	6 (28.6)	-	
Don’t know	20 (1.6)	0 (0.0)	-	
Father’s education				
Illiterate	392 (26.9)	12 (57.1)	-	0.040*
Primary school	359 (24.7)	4 (19.1)	-	
High school	231 (15.9)	1 (4.8)	-	
College/University	131 (9.0)	2 (9.5)	-	
Others ^a^	343 (23.6)	2 (9.5)	-	
Mother’s education				
Illiterate	585 (40.2)	15 (71.4)	-	0.097
Primary school	280 (19.2)	3 (14.3)		
High school	183 (12.6)	1 (4.8)	-	
College/university	35 (2.4)	0 (0.0)	-	
Don’t know	373 (25.6)	2 (9.5)	-	
Flooring type				
Mud	74 (5.1)	0 (0.0)	-	0.018*
Concrete/tile	580 (39.8)	3 (14.3)	-	
Others ^b^	802 (55.1)	18 (85.7)	-	
House type				
Hut	208 (14.3)	2 (9.5)	12.2731	0.007*
Stone house	576 (39.6)	16 (76.2)		
Mud house	181 (12.4)	1 (4.8)		
Modern concrete house	491 (33.7)	2 (9.5)		

* Fisher’s exact

^***a***^ Non-Formal Education, Monastic education, Don’t know

^***b***^ Bamboo, Plank

## Discussion

The present study attempted to assess the prevalence, intensity, and factors related with STH among students in Bhutan.

The result from this study showed that the overall prevalence of any STH infection as well as the prevalence of individual helminths were very low in all three regions. This overall prevalence was lower than the one found in the 2003 study among school children in western Bhutan, with a cumulative prevalence of 16.5%[13]. A similar trend in the drastic reduction of STH prevalence was also observed from 2004 among school-aged children in Nepal[20]. This substantial decrease in the prevalence may have been contributed by the synergistic effect of regular school based deworming, overall improvement in the living standards, sanitation, hygiene and other public health achievements over the years.

Among the different species of STH, *A*. *lumbricoides* and *T*. *trichiura* were more prevalent in this study with less hookworm detection. This finding is consistent with earlier studies [11, 13, 21]. This could be explained by the similar mode of entry into human being through ingestion and similar environmental conditions required for embryonation of these two species. Besides, these two species most intensely infect children of school going age with a decline in adulthood whereas the frequency and intensity of hookworm infection are usually high in adulthood[3].

Region wise, there was no significant difference in the prevalence of STH although the number of infections was slightly higher in the eastern region. This similarity in prevalence could be interpreted in terms of nationwide coverage of school based deworming intervention, similar socio-economic status, improvement in the overall living standards, sanitation, hygiene and other public health interventions, although this needs to be ascertained with further studies.

Very few studies have looked into the comparison of the prevalence of STH infection between urban and rural setting revealing complicated and mixed picture. In our study, no significant difference in the prevalence of STH infection was found between urban and rural settings. Similar findings were also reported from Nepal [20]. In contrast, few studies reported higher prevalence in rural areas[22, 23] while others found higher prevalence in urban areas [24].

We found father’s occupation was associated with STH infection. A higher rate of infection was found in students who reported father’s occupation as farmer compared to other occupational groups. This finding is concordant with other studies [25, 26], which reported higher risk of STH infection in students whose parents were farmer. It is possible that a father, who is usually the head of the family, with low income will be forced to settle in a poor living environment, which is associated with high STH transmission [7].

Significant association was also found between father’s education level and presence of any STH infection. More infection was observed in those students’ who reported their fathers were illiterate compared to other groups. This is in contrast to earlier studies from India and China [21, 27], which reported no association. In general, several studies [27–29] established negative association between mother’s education and STH infection. The finding in our study could be explained in terms of confounding by other socio-economic factors or individual behaviors like personal hygiene practices, maintaining clean living environment, which may be associated with parent’s education.

Similarly, type of houses where students resides and type of flooring in the house were significantly related to any STH infection in this study. Presence of any STH infection was more in students who reported residing in stone houses than in other type of house. The stone houses in this study were similar to huts and were built from stones plastered with mud. Studies elsewhere [21, 24] have reported living in hut as a risk factor for STH infection. A higher rate of infection was also found in students who reported their flooring type as ‘others,’ which mainly consisted of wooden planks or bamboo. Several other studies [15, 24, 27] found floor type was not significantly associated with STH infection after adjusting for household wealth. It was unexpected to see more infection in those with plank flooring, which is considered finished and clean floor. Probably, it could be either the floors were not clean or the effect of potential confounders such as socio-economic status, hygiene and sanitation behavior or overall environmental conditions.

Water, sanitation and hygiene (WASH) practices are important in the prevention of STH transmission [1, 2]. No significant relation was observed between individual WASH items and STH infection in this study. However, many students reported toilets not always kept clean, not washing hands regularly before meal and after defecation, and practice of constant or partial open defecation, which are potential risk factors for STH transmission [21, 28]. Studies elsewhere reported drinking unclean water [27], untrimmed finger nails, and not washing hands before meals [29] as an important correlate of STH infection. This indicated the need for integrated control of STH infection with deworming and improvement in water, sanitation and hygiene practices through health education and facilitating the availability of such provisions are significant.

The present study was conducted to investigate the prevalence of STH and their correlates in Bhutan. However, it had some limitations and potential bias: First, the fact that only students in government schools were investigated. However, the number of private schools in Bhutan is very low and normally the sanitation conditions in private schools are better and therefore, we consider that this bias is not invalidating findings of our study. Second, study design, being cross-sectional, it could not underline the causal effect between the STH infection and other independent factors related to it. Third, we are not able to reach the estimated sample size (1500 children) due to inability to provide specimen or insufficient quantity of stool specimen, thus limiting to 1456 children at the end of study. However, due to big decline in average prevalence (from 16.5 in 2003 to 1.4 in 2017), this sample was sufficient to demonstrate a statistically significant difference from the baseline prevalence. Fourth, the poor sensitivity of mini-FLOTAC method for very light intensity infection [18] and use of single stool specimen might have underestimated the prevalence as egg excretion varies over hours and days [30, 31]. However, great care was taken to maintain high standards in performing the procedures starting from the collection of samples through analysis and recording. Fifth, the report on correlates of STH infection was restricted to the variables measured and may not present a complete picture of the risk patterns. For example, information on family income, vegetable processing, nutritional status, etc. were not collected. Sixth, the questionnaire was not translated to the local dialect and could have created misunderstanding among students in lower grades when responding. In addition, the responses relied on the self-report of the respondents, which may have led to under or over estimation. Lastly, the evaluation of association between potential risk factors and STH infection was limited only to bivariate analysis. Due to very low prevalence, we could not examine mutually adjusted effect of potential risk factors in STH infection.

## Conclusion

In conclusion, the present study showed that the STH epidemiological situation in Bhutan with an initially high STH endemicity and after establishing a successful STH control programme, infection saw decline. A similar decline has been observed in several other countries like Sri Lanka [32], Nepal [20], Afghanistan [33], Vietnam [34], Myanmar [35] and Lao PDR [36]. WHO estimated that preventive chemotherapy intervention will globally avert in children over 900000 Disability Adjusted Life Years(DALYs) in 2020 [37]. This intervention when applied to women of reproductive age decreases anaemia and improve birth-weight [38]. In Bhutan, despite the very low STH prevalence, the questionnaire administered in the study showed that the sanitation situation is not ideal in the country and environmental contamination with human faeces is still present with almost 68% of the students reporting constant or partial open defecation. For this reason, there is a risk, if the control programme is interrupted, that the STH epidemiology will return to the original level of high prevalence and intensity. Therefore, in line with WHO recommendation, confirmed by recent reviews on 15 STH endemic countries [39], it is appropriate to reduce the frequency of drug administration to once a year, maintain surveillance system to early identify possible prevalence rebound, and focus activities on concerted health education and improvement appropriate sanitation practices.

## Supporting information

S1 Dataset Supporting data set for which statistics are computed. (DTA)

S1 Text Ethics board approval letter. (PDF)

S2 Text Survey questionnaire. (DOCX)
