# Supplementary material for: Soil-Transmitted Helminth infections reduction in Bhutan: A report of 29 years of deworming
Source: PLoS One. 2020 Jan 3;15(1):e0227273. doi: 10.1371/journal.pone.0227273 (PMC6941809; doi:10.1371/journal.pone.0227273)
Supplement: S1 Text — (PDF) [file pone.0227273.s002.pdf]

དཔལ་ལྡན་འབྲུག་གཞུང་།
གསོ་བ་ལྷན་ཁག
གསོ་བའི་ ཞིབ་འཚོལ་ ཀུན་སྤྱོད་རྣམ་གཞག་ བཀོད་ཚོགས།
ཐིམ་ཕྱུག

ROYAL GOVERNMENT OF BHUTAN
MINISTRY OF HEALTH
RESEARCH ETHICS BOARD OF HEALTH
THIMPHU : BHUTAN
P.O. BOX : 726

REBH/Approval/2017/033 **12th April, 2017**

REBH Approval Letter

**PI:** Mr. Tshering Dukpa<br>**Institute:** Faculty of Nursing and Public Health(FNPH), KGUMSB, Thimphu	**Study Title**:<br>Prevalence and Intensity of Soil Transmitted Helminthiasis among School Children in Bhutan
**Co-Investigators**: 1. Dr. Nidup Dorji; 2. Dr. Diki Wangmo; 3. Mr. Wangchuk Dukpa; 4. Mr. Sangay Thinley; 5. Ms. Passang Lhamo; 6. Mr. Karma Tshering; 7. Ms. Kinley Gyem 8. Dr. Palden Wangchuck; and 9. Mr. Tshering Dorji	
**Mode of Review**: ✓Full board review (Meeting no. 01/2017-32nd) for version 1 and Expedite review for version 2 and 3	
**Decision**: Approved **(Note: Abide by the conditions of approval)**	
**Conditions for Approval**<br>1. *This approval is granted for the scientific and ethical soundness of the study. The PI shall be responsible to seek all other clearances/approvals required by law/policy including permission from the study sites before conducting the study.*<br>2. *Report serious adverse events to REBH within 10 working days after the incident and unexpected events should be included in the continuing review report or the final report.*<br>3. *Any changes to the proposal or to the attachments (informed consent and research tools such as forms) should be approved by REBH before implementation.*<br>4. *Final report of the study should be submitted to REBH at the end of the study for protocol file closure.*<br>5. *This approval is valid through 11/04/2018. If the study has to continue beyond the approved period the PI has to apply for the continuing review two months before the validity of the approval expires.*	

(Dr. Tashi Tobgay)
Chairperson

For further information please contact: *REBH Member Secretary, msgurung@health.gov.bt/tashidema@health.gov.bt*: *Tel: +975-2-322602 ext 333*
