# Supplementary material for: Soil-Transmitted Helminth infections reduction in Bhutan: A report of 29 years of deworming
Source: PLoS One. 2020 Jan 3;15(1):e0227273. doi: 10.1371/journal.pone.0227273 (PMC6941809; doi:10.1371/journal.pone.0227273)
Supplement: S2 Text — (DOCX) [file pone.0227273.s003.docx]

**Annexure I**

**Survey Questionnaires (Version 2.0)**

***Prevalence and Intensity of Soil Transmitted Helminth among School Children in Bhutan***

Participant No: Date of Interview: Name of Interviewer:

**A. School Information**

| 1. Name: |  |
| --- | --- |
| 2. Dzongkhag: |  |
| 3. Gewog: |  |
| 4. Chiwog: |  |
| 5. Setting (please tick [✓] one only) | Rural  Urban |

| 6. Level of School  (Please tick [√] one only) |
| --- |
| Lower Secondary School |
| Middle Secondary School |
| Higher Secondary School |
| Central School |

**B. Socio –demographic Information**

Female…….1

male……….2

1. Student Name: 2. Age (in years): 3. Sex:

Boarding

Day Scholar

4. Grade (Please tick [✓] one only): 5. Type of Student: (Please tick [✓] one only

III  IV  V

VI  VII  VIII

Farmer

Civil Servant

Corporate employee

Private employee

Others (Please specify:…………………

………………………………………………

6. Ethnicity (Please tick [✓] one only): 7. Father’s Occupation

Ngalop

Sharchop

Lhotshamp

Kheng- Bumthap

Ohers (Please specify:…………………...

……………………………………………….

Illiterate

Non- Formal Education

Primary Level

High School

College/ University

Others (Please specify:……………………

…………………………………………………

Farmer

Civil Servant

Corporate employee

Private employee

Others (Please specify:……………

……………………………………………

8. Mother’s Occupation 9. Father’s Educational Level

Hut *(Bago)*

Stone House *(Tsikchim)*

Mud House *(Sachim)*

Modern Concrete House

Others *(Please specify:…………………….*

*……………………………………………………..*

10. Mother’s Education Level 11. What is the type of house you live in?

Illiterate

Non- Formal Education

Primary Level

High School

College/ University

Others (Please specify:………………

…………………………………………

12. How many stories/floors does your 13. What is the type of flooring used in your

Mud *(earth)*

Concrete *(cement)*

Tiles

Others *(Please Specify:………………….*

*…………………………………………………...*

One storied

Two storied

Three or more storied

house have? house?

**C. Deworming History**

1. In the last six months, have you used deworming tablet?

Yes

No

**D. Behavioral Risk Factors (Water, Sanitation and Hygiene [WASH] Behavior of the**

**student)**

| **Item No.** | **Behavioral WASH Items** | **Always** | **Sometimes** | **Never** |
| --- | --- | --- | --- | --- |
| **Water** | | | | |
| 1 | How often do you drink tap water? |  |  |  |
| 2 | How often do you drink water from other sources such as river, stream? |  |  |  |
| 3 | How often do you drink collected rainwater? |  |  |  |
| 4 | How often do you drink water from the pond? |  |  |  |
| 5 | How often do you drink boiled water? |  |  |  |
| 6 | How often do you drink filtered water? |  |  |  |
| **Sanitation** | | | | |
| 7 | Are toilets in school clean? |  |  |  |
| 8 | How often do you use flush toilet for defecation? |  |  |  |
| 9 | How often do you use pit latrine for defecation? |  |  |  |
| 10 | How often do you practice open defecation  (field, bushes, drain)? |  |  |  |
| **Hygiene** | | | | |
| 11 | Is there water available for hand washing at schools? |  |  |  |
| 12 | Is there soap available for hand washing at schools? |  |  |  |
| 13 | How often do you wash hands before meals? |  |  |  |
| 14 | How often do you wash hands with soap? |  |  |  |
| 15 | How often do you wash hands after defecation? |  |  |  |
| 16 | Do you carryout agricultural works at home/school? |  |  |  |
| 17 | How often do you put footwear outside the house? |  |  |  |
| 18 | How often do you wash fruits before consuming? |  |  |  |
| 19 | How often do you use spoon for eating? |  |  |  |
| 20 | Do you keep your nails short and clean? |  |  |  |
